# Mindsets matter for every patient and we can all help

**DOI:** 10.1192/bjo.2023.524

**Published:** 2023-07-20

**Authors:** David Ring

**Affiliations:** Dell Medical School, University of Texas at Austin, Austin, Texas, USA

**Keywords:** Stigma and discrimination, anxiety- or fear-related disorders, cognitive–behavioural therapies, depressive disorders, psychosocial interventions

## Abstract

People seek care when a sensation becomes a symptom (a concern). Levels of discomfort and incapability are associated with feelings of distress or unhealthy misinterpretation. To limit mental health stigma, it is important to emphasise that this is about how the human mind works (mindsets) and not just about mental illness. Experts in mental health and in pathophysiology can work together, each doing their part to optimise mindset.

The evidence that mindsets and circumstances are key contributors to levels of discomfort and incapability is strong.^1–4^ Now we are working to implement these facts into everyday care.^5^ The rationale for addressing mindsets along with physical symptoms is straightforward. The human mind is constantly interpreting,^6^ and the autopilot part of the mind is programmed to prepare for the worst. Patients present with a sensation that has become a symptom, meaning the sensation has become a concern.^7^ Consequently, we can be on the lookout for misconceptions, even small ones, because they are so common.

There is evidence that common misconceptions about sensations are more fixed and impactful the greater one's level of distress (feelings of anxiety and depression).^8,9^ Some small or large feelings of worry or despair are experienced by humans every day. And these feelings are important even if they do not reach a threshold level for application of a diagnosis. In other words, mental health is continuous, not dichotomous. And all of us can strive for a healthier mindset. Dichotomising mental health as mental illness, or a mental health diagnosis, is a false dichotomy that reinforces mental health stigma. Co-management of diagnosed mental health and musculoskeletal conditions is one option, often referred to as collaborative care.^10^ An alternative, perhaps more suitable approach might be the crafting of care strategies (outlined herein) that treat the whole person. Every person, every time.

## The health benefits of accommodation

Everyone over age 50 has at least one musculoskeletal disease and some level of sensations from that disease. For instance, most of us develop osteoarthritis of the trapeziometacarpal^11^ and knee joints^4^ and rotator cuff tendinopathy^12^ as we age. It is important to adequately distinguish the following: people with a disease (pathophysiology), people who notice sensations from the disease, people who become concerned about what the sensations signal about their health (people with symptoms) and people concerned enough about the symptoms to seek care. Musculoskeletal care is largely discretionary and infrequently necessary. Those of us who see a specialist are usually satisfied with a single visit.^13^ In other words, accommodation is an effective and highly utilised health strategy. The key aspects of accommodation may be healthy mindsets and healthy circumstances (security in employment, finances, home, food, relationships and roles).^14^

The importance of mental and social health in musculoskeletal illness can be seen in the marked discordance of levels of disease (pathophysiology severity) and levels of illness (the state of being unwell – discomfort and incapability).^2,15^ People with musculoskeletal pathology are less comfortable and less capable in proportion to symptoms of depression and anxiety and levels of unhelpful thinking such as worst-case thinking and fear of painful movement.^1,3^

## Comprehensive whole-person care

The trial proposed by Teixeira et al^10^ will measure the feasibility and acceptability of adding a case manager to prioritise alleviation of symptoms of worry and despair as part of non-operative musculoskeletal out-patient care. That goal will be served not by diagnosing people with anxiety or depression, but rather by anticipating some level of distress and strategising care so it alleviates that distress, and by anticipating misinterpretation of sensations and making sure the care identifies and gently reorients those misconceptions.

I enthusiastically support the strategy of leveraging the average person's high regard for physical, occupational and hand therapy and incorporating aspects of mindset training into their care. The combination of physical and mental training (variations of cognitive–behavioural therapy) is referred to as psychologically informed physical therapy^16^ or cognitive functional therapy.^17^ Every member of the care team can be attuned for common misconceptions and feelings of distress in every patient. And the way we address symptoms can be constructed to promote healthy mindsets.

## Data Availability

Data availability is not applicable to this article as no new data were created or analysed in this study.
